# Multi-band Metasurface-Driven Surface-Enhanced Infrared Absorption Spectroscopy for Improved Characterization of in-Situ Electrochemical Reactions

**DOI:** 10.1021/acsphotonics.3c01592

**Published:** 2024-01-26

**Authors:** Malo Duportal, Luca M. Berger, Stefan A. Maier, Andreas Tittl, Katharina Krischer

**Affiliations:** † Department of Physics, Technical University of Munich, Garching 85748, Germany; ‡ Chair in Hybrid Nanosystems, Nanoinstitute Munich, and Center for NanoScience, Faculty of Physics, Ludwig-Maximilians-University Munich, Königinstraße 10, München 80539, Germany; § School of Physics and Astronomy, Monash University, Clayton, VIC 3800, Australia; ∥ Department of Physics, Imperial College London, London SW7 2AZ, United Kingdom

**Keywords:** nanophotonics, metasurfaces, surface-enhanced infrared absorption spectroscopy, electrochemical CO_2_ reduction, in situ spectro-electrochemistry

## Abstract

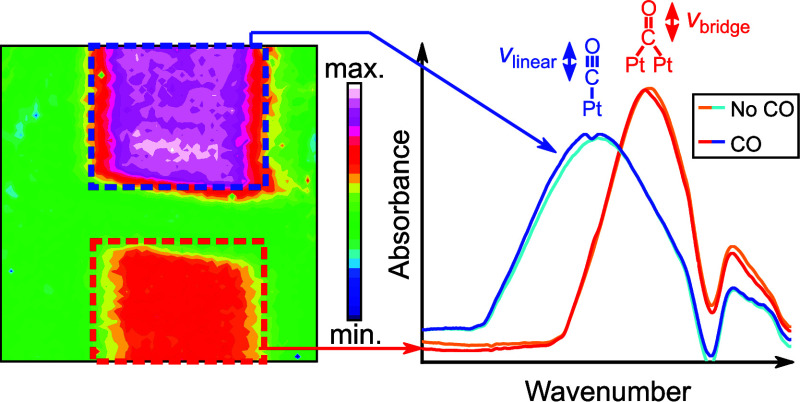

Surface-enhanced spectroscopy techniques are the method-of-choice to characterize adsorbed intermediates occurring during electrochemical reactions, which are crucial in realizing a green and sustainable future. Characterizing species with low coverage or short lifetimes has so far been limited by low signal enhancement. Recently, single-band metasurface-driven surface-enhanced infrared absorption spectroscopy (SEIRAS) has been pioneered as a promising technology to monitor a single vibrational mode during electrochemical CO oxidation. However, electrochemical reactions are complex, and their understanding requires the simultaneous monitoring of multiple adsorbed species in situ, hampering the adoption of nanostructured electrodes in spectro-electrochemistry. Here, we develop a multi-band nanophotonic-electrochemical platform that simultaneously monitors in situ multiple adsorbed species emerging during cyclic voltammetry scans by leveraging the high resolution offered by the reproducible nanostructuring of the working electrode. Specifically, we studied the electrochemical reduction of CO_2_ on a Pt surface and used two separately tuned metasurface arrays to monitor two adsorption configurations of CO with vibrational bands at ∼2030 and ∼1840 cm^–1^. Our platform provides a ∼40-fold enhancement in the detection of characteristic absorption signals compared to conventional broadband electrochemically roughened platinum films. A straightforward methodology is outlined starting with baselining our system in a CO-saturated environment and clearly detecting both configurations of adsorption. In contrast, during the electrochemical reduction of CO_2_ on platinum in K_2_CO_3_, CO adsorbed in a bridged configuration could not be detected. We anticipate that our technology will guide researchers in developing similar sensing platforms to simultaneously detect multiple challenging intermediates, with low surface coverage or short lifetimes.

## Introduction

The study of intermediates occurring during electrochemical reactions remains a challenge due to low surface coverage and short lifetimes. Raman and IR spectroscopy are powerful in situ optical characterization techniques that can detect the rotational or vibrational modes of molecules. During the CO_2_ reduction reaction (CO_2_RR), a key intermediate is adsorbed CO, which is detectable with IR spectroscopic techniques.^1^ However, as IR characterization techniques suffer from high losses by aqueous electrolytes, two specific geometries that minimize the light path through the electrolyte are typically used to acquire the IR spectra of molecules adsorbed on electrode surfaces. First, the external reflectance approach consists of squeezing a thin layer of electrolyte between the working electrode and a prism to reduce the optical losses.^2,3^ The advantage of the external reflectance approach is a high degree of freedom in choosing the morphology and thickness of the studied material. However, although the electrolyte layer is thin, it still suffers from dampened signals due to light absorption from the electrolyte. Moreover, the need for a thin electrolyte layer strongly limits diffusional processes. This makes the external reflectance approach an inadequate technique for in situ studies of electrocatalytic reactions, such as the CO_2_RR.

A second geometry used to decrease optical losses from the electrolyte is to operate a sensing platform in an attenuated total internal reflection (ATR) configuration.^2−4^ As light is totally reflected on a surface, evanescent waves with exponential decay probe the other side of the reflective surface. Due to the evanescent approach, the losses stemming from the electrolyte are minimized. This so-called Kretchmann configuration leaves the electrode freely accessible for transport processes to and from the electrolyte. However, it requires thin-film electrodes as the quickly decaying evanescent waves must be able to penetrate the electrolyte past the metal/electrolyte interface to probe the analyte, but as the metal layer is decreased in thickness, the electrochemical stability decreases.^3^ In addition, ATR infrared absorption spectroscopy at the plane electrodes yields only low IR signals. This disadvantage is overcome when rough film electrodes are prepared, for example, by electrochemical roughening. Then, the electron density of nanoparticles with a linear dimension of the order of the wavelength of IR irradiation can come into resonance with the electromagnetic wave, leading to an enhanced vibrational signature.^5^ The latter methods is usually referred to as ATR surface-enhanced infrared absorption spectroscopy or, in short, ATR-SEIRAS. However, ATR-SEIRAS introduces challenges in both film electrode stability and reproducibility of the acquired spectra. The intensity of the vibrational bands strongly depends on the distribution of the nanoparticle size, resulting in an uncontrolled and random signal enhancement, making this technique difficult to reproduce and often unreliable.^6^

Recently, a promising single-band nanophotonic-electrochemical platform based on ATR-SEIRAS was designed, employing a nanostructured platinum surface to characterize CO during CO oxidation.^7^ The precisely nanostructured platinum metasurface that integrated SEIRAS with cyclic voltammetry for the study of electrochemical interfaces provided a clear and reproducible method to produce SEIRAS-active electrodes. Specifically, the electric near-field enhancement produced by the platinum-based nanoslot metasurface was shown to amplify the in situ-generated signal traces of the vibrational mode of linearly adsorbed CO (CO_linear_) at ν_linear_ = 2033 cm^–1^. Changes in the reflection intensity based on the coupling of a metasurface-driven resonance and the vibrational mode of an adsorbed species controllably enhanced its characteristic signal trace. However, the reported nanophotonic-electrochemical platform featured only one resonance and could therefore only spectrally target and enhance one vibrational mode. Since electrochemical reactions are complex and their understanding requires the simultaneous in situ monitoring of multiple adsorbed species, the use of single-band metasurface-driven SEIRAS in electrochemistry was hampered. At the same time, a methodology for reproducible and signal-enhancing working electrodes is required to resolve adsorbed species with weak signals, fast conversion, and low surface concentrations. To the best of our knowledge, a platform that offers high resolution, reproducible fabrication of the working electrode, and simultaneous monitoring of multiple adsorbed species has not yet been reported in the literature.

**Figure 1:**
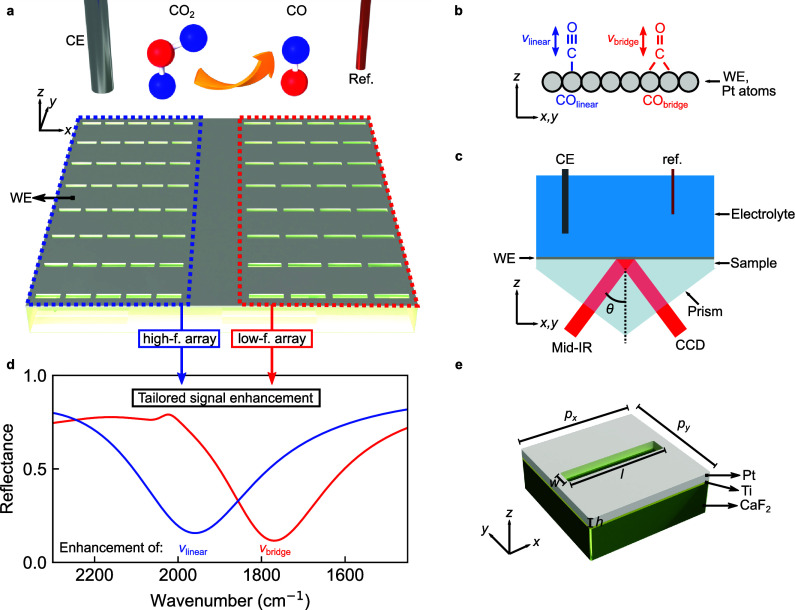
Concept and numerical design of the multiband nanophotonic-electrochemical platform. (a) Schematic of the multi-band platinum-based nanoslot metasurface to study in situ CO_2_ reduction. The metasurface contained a high-frequency (high-*f*) and low-frequency (low-*f*) array to monitor the signals of CO_linear_ and CO_bridge_, respectively. (b) Schematic showing the chemical structure of the two adsorption configurations of CO on platinum. (c) Schematic illustrating the metasurface in an electrochemical chamber filled with electrolyte that was illuminated from below in an ATR geometry. (d) Resonances stemming from two metasurface arrays on Pt. The shared geometrical parameters were *h* = 30 nm, *w* = 180 nm, *p*_*y*_ = 1420 nm, and *p*_*x*_ – *l* = 230 nm with the slot length being swept from (blue) *l* = 1370 nm to (red) *l* = 1580 with the parameters defined in (e) showing a sketch of the unit cell. A 1 nm-thick Ti adhesion layer was utilized in the structure’s fabrication for improved adhesion of Pt on CaF_2_.

Here, we develop multi-band metasurface-driven SEIRAS for the improved characterization of in situ electrochemical reactions (Figure 1a). We demonstrate its successful operation during the CO_2_RR where the molecular signals of two configurations of adsorption of CO on platinum are resolved: on-top (CO_linear_) and bridge-bound (CO_bridge_) CO molecules, respectively (Figure 1b). So far, little attention has been given to the involvement of bridge site configurations of CO on Pt during the CO_2_RR. The few studies that have been conducted focused on acidic media.^8−11^ A fundamental and systematic study of bridge site configurations during the CO_2_RR in alkaline media is still missing. We use a platinum nanoslot metasurface on a CaF_2_ substrate featuring two arrays that were each numerically modeled and tuned to spectrally target one of the two aforementioned characteristic molecular vibrations of the CO_2_RR. For each vibrational mode, a unique array with a spectrally targeted resonance was fabricated. Each array locally enhanced the electric near-fields and enhanced the corresponding molecular signal traces. Our two-band approach can be extended to multiple vibrational bands.

SEIRAS was performed in an ATR geometry (Figure 1c) to maintain free accessibility of the electrode surface and minimize the contribution of the electrolyte to the IR spectrum. We validated the nanophotonic-electrochemical platform by following the oxidation of CO into CO_2_ during a cathodic polarization, measuring simultaneously the top and bridge site adsorptions of CO. The clear detection and enhancement of the top and bridge adsorption configurations of CO on Pt were confirmed by observing the typical Stark shift in the molecular signal traces. The vibrational bands of CO_linear_ and CO_bridge_ were located at a ν_linear_ of ∼2030 cm^–1^ and a ν_bridge_ of ∼1840 cm^–1^, respectively. Then, we followed the reduction of CO_2_ in situ during which we recorded a strong CO_linear_ signal. Despite the high resolution achieved with our nanophotonic-electrochemical platform, the CO_bridge_ was not detected during the CO_2_RR. Finally, we established a methodology to implement similar multi-band nanophotonic-electrochemical platforms, providing a framework for future research in this area.

## Experimental Section

### Numerical Simulations

In this study, the optical properties of our multiband nanoslot metasurface was numerically modeled with CST Studio Suite (2021) with the finite-element frequency-domain Maxwell solver. For the simulation of CaF_2_, a refractive index of 1.4 was used, while the surrounding medium was represented by water with a refractive index of 1.33. Platinum was modeled using its experimental complex refractive index data.^12^ To introduce linearly polarized light at an angle of incidence of 72° into the system, an impedance-matched open port with a perfectly matched layer was employed. Since light experiences total internal reflection at this angle at the CaF_2_–Pt interface, the boundary opposite the open port was set as a perfect electric conductor. The unit cell was defined and simulated as an infinite periodic array using Floquet boundaries.

### Analytical Analysis of Resonances

The resonances were characterized in terms of their radiative (γ_rad_) and intrinsic (γ_int_) damping rates from which their total quality factor could be determined as , where ν_0_ is the central wavenumber. We employed temporal coupled mode theory^13^ according to ref (14), describing a resonator with a single port that supported reflected waves and a single resonance that coupled to the far field via the coupling constant . Additionally, an intrinsic loss channel introduced damping to the resonance at a rate of γ_int_. The reflectance spectra (*R*) were fitted by
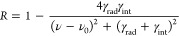
where ν is the wavenumber.

### Multi-band Metasurface Fabrication

The multi-band metasurface fabrication was similar to the protocol provided in ref (7). Instead of one array, two arrays were fabricated ∼500 μm apart to fit within the window of the focal plane array of the IR spectrometer. The arrays were each approximately 3 × 2 mm in size, showing that large arrays could be made. The arrays had to be large as the Fourier transform IR spectrometer did not have and did not need a focusing microscope objective. We chose CaF_2_ as the substrate due to its transparent properties within the mid-IR spectral range as well as its high chemical stability and low solubility. Before the experiments, the substrate underwent a thorough cleaning process involving an acetone bath in an ultrasonic cleaner followed by an oxygen plasma treatment. Subsequently, the substrate was spin-coated with an adhesion promoter (Surpass 4000) followed by a layer of negative-tone photoresist (ma-N 2403). The photoresist was baked at 100 °C for 60 s, and a conducting layer (ESpacer 300Z) was deposited using spin-coating. The metasurface patterns were generated by defining a unit cell and replicating it in both the *x* and *y* directions. Electron-beam lithography (Raith Eline Plus) was employed to write the patterns using an acceleration voltage of 30 kV and an aperture of 20 μm. The exposed resist was developed in ma-D 525 for 70 s at room temperature. Thereafter, a titanium adhesion layer (1 nm at 0.4 Å s^–1^) and a platinum film (30 nm at 2 Å s^–1^) were deposited on the patterned surface using electron-beam evaporation (PRO Line PVD 75, Lesker). Finally, the fabrication process was completed with an overnight lift-off process in mr-REM 700. For comparison, a pure 30 nm-thick platinum film on a 1 nm titanium layer on CaF_2_ was utilized as a reference.

### Surface-Enhanced Infrared Absorption Spectroscopy Measurements

**Figure 2:**
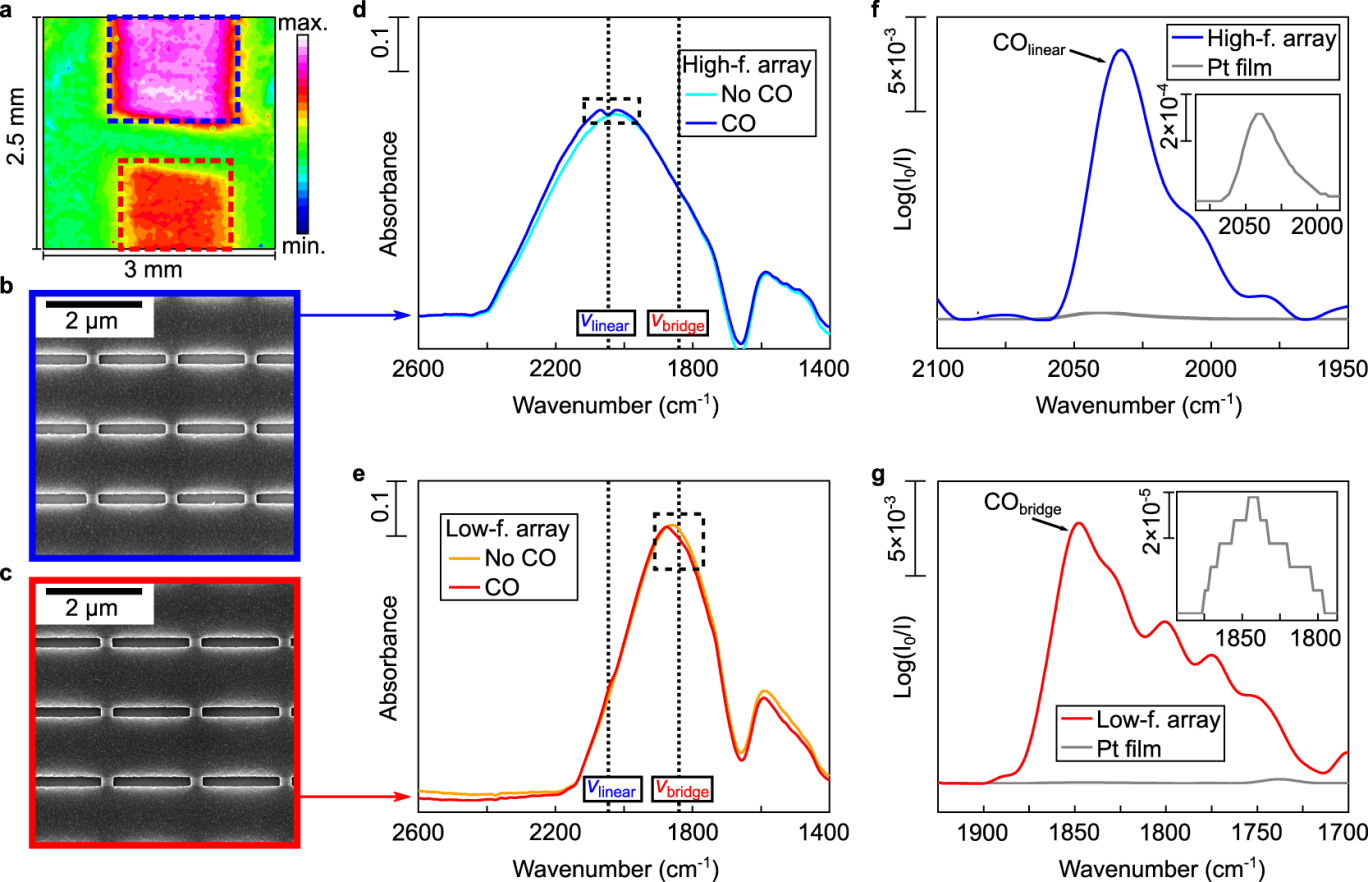
Characterization of the multiband nanoslot metasurface. (a) Heat map of the metasurface obtained by integrating the SEIRAS signal from 1600 to 2800 cm^–1^. (b, c) SEM pictures of the (b) high and (c) low-frequency arrays indicated via blue and red dashed boxes, respectively. (d, e) Resonances with and without CO for the (d) high and (e) low-frequency arrays in 0.5 M K_2_CO_3_ at the OCP. The spectral positions of CO_linear_ (ν_linear_) and CO_bridge_ (ν_bridge_) are indicated. (f, g) Comparison of the differential absorbance obtained with the (f) high and (g) low-frequency array with an unstructured Pt film (30 nm-thick, inset) and with the multi-band nanoslot metasurface at the OCP.

SEIRAS was conducted using a Vertex 80 spectrometer equipped with an IMAC Focal Plane Array macro imaging accessory from Bruker. A specular reflection unit (VeeMax III from PIKE Technologies) paired with a CaF_2_ prism and light polarizer introduced light at 72° with respect to an electrochemical jackfish cell, thereby enabling the attenuated total internal reflection geometry. A focal plane array detector (64 × 64 MCT detectors) was used to characterize the optical properties of the multiband nanoslot metasurface. By integrating the measured (absorbance) signal across the spectral range corresponding to the metasurface-driven resonances (1600–2800 cm^–1^), a pixelated two-dimensional heat map of the sample was created to identify the pixels corresponding to the nanostructured arrays (Figure 2a). Then, the pixels corresponding to each array were averaged to construct the final spectra. The samples were cleaned via electrochemical cycling using a scan rate of 20 mV s^–1^ from 0 to 1600 mV_RHE_. Prior to each measurement, an initial background was acquired using p-polarized light. The metasurface-driven resonances were measured in situ by using s-polarized light. Each spectrum was acquired at a resolution of 4 cm^–1^ and by averaging 10 scans. The data was treated by applying a baseline correction and Savitzky-Golay filter. The enhancement provided by the multiband metasurface was determined by comparing the area of the peaks associated with the vibrational modes of the CO_linear_ and CO_bridge_ configurations to the corresponding measurements performed on an unstructured Pt film (30 nm).

### Electrochemical Measurements

A classical three-electrode system was implemented using the Pt multiband metasurface as the working electrode, a Pt wire as the counter electrode, and a saturated calomel electrode (*E* = 0.244 V_RHE_) as the reference electrode. The electrolyte was a 0.5 mol·L^–1^ K_2_CO_3_ solution, saturated with either Ar, CO (both pH 11.9), or CO_2_ (pH 8). Before the first characterization, the cleanliness of the electrode surface was verified by performing a cyclic voltammogram at a scan rate of 20 mV s^–1^ in the Ar-saturated electrolyte.

The behavior of the nanoslots was first characterized by comparing the metasurface-driven resonances in Ar and CO-saturated electrolyte at the OCP. After bubbling CO for 2 h, cyclic voltammetry was performed from +1650 mV_RHE_ to −85 mV_RHE_ using a scan rate of 0.25 mV s^–1^.

Finally, we saturated the electrolyte with CO_2_, which decreased the pH to approximately 8. After 2 h of gas bubbling, cyclic voltammograms were measured with a slow scan rate of 0.25 mV s^–1^ from the OCP (around 650 mV_RHE_) to +1425 mV_RHE_ and then back to +25 mV_RHE_. SEIRAS spectra were acquired at intervals of 100 mV.

## Results and Discussion

### Numerical Design of a Catalytic Multi-band Nanoslot Metasurface

We start the implementation of our catalytic multiband nanoslot metasurface by defining the fundamental unit cell for the numerical simulations, consisting of a solitary slot within a continuous platinum film on CaF_2_ immersed in water (Figure 1e). The parameters of the unit cell of the nanoslot metasurface are defined in Figure 1e, where *p*_*x*_ and *p*_*y*_ are the unit cell lengths in *x* and *y*. The geometrical parameters of the unit cell can be changed to tune the resonance strength and position of the metasurface. As the goal of our investigations was multiband signal enhancement, the unit cell parameters were separately tuned to two resonance frequencies matching the vibration frequencies of two different adsorption configurations of CO on platinum. Specifically, the goal was a straightforward approach to creating two adjacent nanostructured arrays on platinum that would each enhance one of the two vibrational signals.

A common approach to achieve this is to scale all geometrical dimensions of the system at the same time, that is, used, for example, in biosensing^15^ and catalysis.^16^ However, constraints in the fabrication with negative resists limited the unit cell length in *y* to a minimum of 1.4 μm due to proximity effects. Proximity effects arise due to the scattering of electrons in the resist and substrate due to exposure to an electron beam.^17^ We found that the slots merged, and the quality of the lift-off procedure decreased as the unit cell length in *y* was decreased below 1.4 μm. Therefore, we simplified the resonance tuning protocol, allowing only the slot length to change and considering a shift of ∼80 cm^–1^ between simulation and experiment. By increasing the slot length from 1370 to 1580 nm while leaving all other parameters unchanged, the metasurface-driven resonance was red-shifted by ∼150 cm^–1^ (Figure 1d), corresponding to the spectral separation of the two CO vibrational modes.

The Rayleigh anomaly was tuned to the higher frequency side of the resonance to ensure an optimal sensing performance. To improve the characterization of the resonances, the fitting model was adapted specifically for total internal reflection (see the Experimental Section). The quality factor and coupling ratio γ_ext_*/*γ_int_ were obtained by fitting the simulated resonance in reflection using temporal coupled-mode theory (see the Experimental Section). Based on our simulations, the multi-band nanoslot metasurface achieved a modulation of ∼84 and ∼88% in reflection, a quality factor of 4.3 and 5.0, and a ratio of external to intrinsic coupling of 2.6 and 2.1 for the higher and lower frequency resonances, respectively. Our system can be further optimized by maximizing the modulation in reflection or absorption to push it toward its critical coupling condition.

### Multi-band Metasurface Characterization

According to the literature, the vibrational modes of CO_linear_ and CO_bridge_ are expected to occur at ∼2050 cm^–1^^18−22^ and ∼1850 cm^–1^,^18,19,21,23^ respectively. First, we characterized the optical properties of our multi-band metasurface in electrolyte saturated with Ar and CO using SEIRAS. An example of the heat map obtained in Ar-saturated electrolyte by integrating the IR spectra collected by the focal plane array detector is provided in Figure 2a. The two pink areas correspond to the two metasurface arrays designed to enhance CO detection. The quality of the fabricated slots was verified by scanning electron microscopy images (Figure 2b,c). The signal received by the high-frequency array (Figure 2a, bottom array) was averaged, resulting in a resonance spectrally positioned at ∼2030 cm^–1^ in the Ar-saturated electrolyte (Figure 2d). On the other hand, the average signal from the low-frequency array (Figure 2a, right array) produced a resonance located at ∼1840 cm^–1^ (Figure 2e). In both cases, the system showed a near critically coupled behavior between the metasurface-driven resonances and the vibrational modes of CO,^7^ leading to a dip in the high and low-frequency resonances at 2046 and 1848 cm^–1^, respectively. To extract the signal of the vibrational modes of CO_linear_ and CO_bridge_ more clearly, the reflectance spectra with (*R*) and without (*R*_0_) (obtained at a potential where CO was oxidized) adsorbed CO were converted into their differential absorbance, , to separate the metasurface-driven resonance from the CO signals (Figure 2f,g). Furthermore, a comparison of the differential absorbance in the regions of CO_linear_ and CO_bridge_ obtained on an unstructured Pt film (30 nm-thick) and with our multi-band nanoslot metasurface indicates that the high-frequency array exhibited a signal enhancement of ∼40. This signal enhancement is higher than the enhancement reported in our previous work,^7^ which is attributed to an improved lift-off procedure. The signal enhancement provided by the low-frequency array cannot be reliably estimated as the signal of CO_bridge_ was not clearly distinguishable on the unstructured Pt film. The signal of CO_bridge_ could only be observed here with the multiband metasurface due to its high signal-enhancing properties.

### Behavior in CO-Saturated Electrolyte

**Figure 3:**
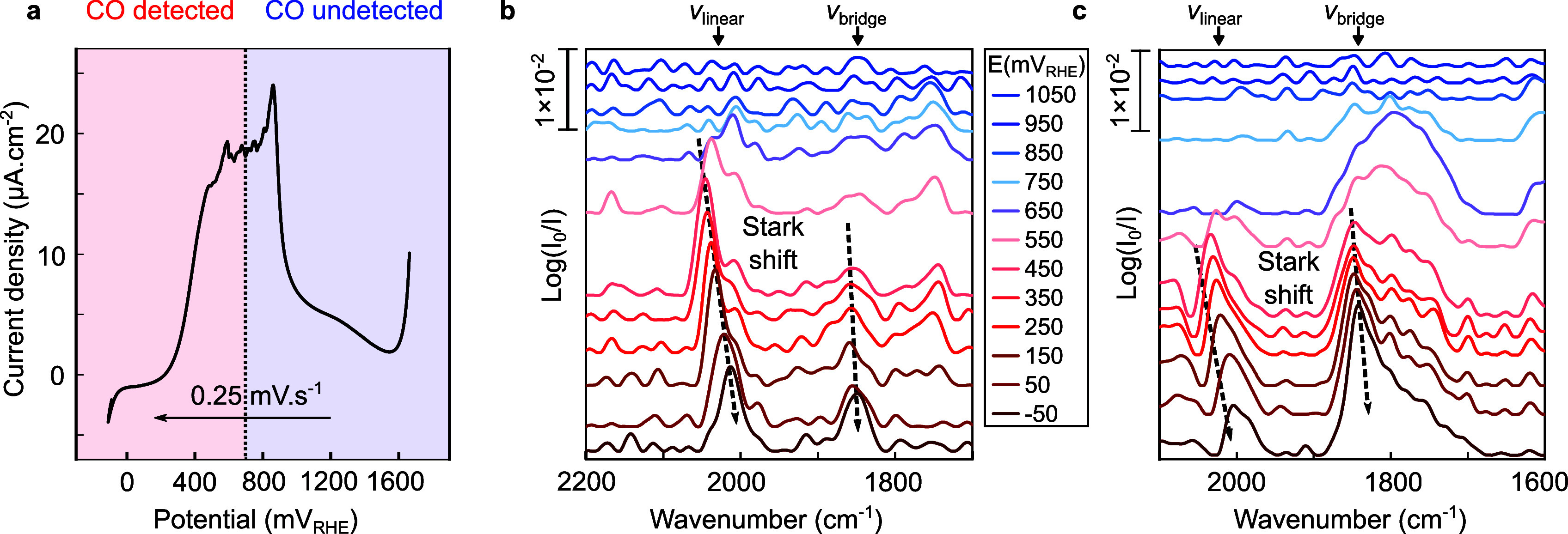
Cathodic polarization of the multi-band metasurface in 0.5 M K_2_CO_3_ saturated with CO. (a) Evolution of the current density during cathodic polarization at 0.25 mV s^–1^. (b, c) Evolution of the IR spectra with the potential acquired by the arrays optimized for (b) CO_linear_ and (c) CO_bridge_ detection. The spectral positions of CO_linear_ (ν_linear_) and CO_bridge_ (ν_bridge_) are indicated.

As a proof-of-concept, the behavior of the multiband nanoslot metasurface in 0.5 M K_2_CO_3_ saturated with CO is provided here using electrochemical voltammetry. A cathodic scan was conducted at a rate of 0.25 mV s^–1^, starting at 1650 mV_RHE_, as shown in Figure 3a. As already observed in the literature,^7,24,25^ the increase in the current around 950 mV_RHE_ followed by a plateau corresponds to the oxidation of CO into CO_2_, which is limited by CO diffusion from the bulk of the electrolyte to the Pt surface. Then, the following decrease in the current at 350 mV_RHE_ indicates the end of the region where CO was oxidized. Finally, the slight drop in the current around 0 mV_RHE_ can be attributed to the hydrogen evolution reaction. SEIRAS spectra were acquired at intervals of 100 mV during the cathodic scan. The potential regions in which CO was or was not detected are shown as red and blue regions, respectively.

The evolution of the IR spectra with the electrical polarization of the high (Figure 3b) and low-frequency (Figure 3c) arrays shows the successful detection of the vibrational signals of CO_linear_ and CO_bridge_, respectively. The vibrational modes appeared as peaks in the differential absorbance spectra. Regarding the behavior of CO_linear_, a spectral shift of approximately 63 cm^–1^ V^–1^ was observed from 450 to −50 mV_RHE_, which is in line with the literature^7,26−29^ and can be attributed to either a higher π-back-donation from the metal to CO^27,30^ with increasing potential and/or the Stark shift.^28,30,31^ Additionally, a significant spectral redshift attributed to the decrease of the dipole–dipole interactions as the coverage decreases^32,33^ was resolved from 650 to 450 mV_RHE_, owing to the high resolution achieved with our platform. Concerning the behavior of CO_bridge_ on the low-frequency array, a distinct peak was resolved, exhibiting similar behavior to CO_linear_. However, the observed Stark shift in this case was smaller, resulting in a value of approximately 21 cm^–1^ V^–1^. This difference in Stark shift between CO_linear_ and CO_bridge_ has been observed in the literature.^28,29^ However, the origin of this difference is still controversial as other authors suggested that both configurations should provide the same Stark shift.^34,35^ Our observations can be explained by the smaller IR cross-section of CO_bridge_ compared to CO_linear_.^34^ When CO is adsorbed linearly on the platinum surface, it strongly binds to the Pt atoms,^36^ which could explain the larger Stark shift. Furthermore, the electric field from the metal surface could have affected CO_linear_ more significantly, leading to a larger change in its vibrational frequency. On the other hand, the configuration of CO_bridge_ may have resulted in a weaker interaction with the metal surface.^36^ In that case, the electric field from the metal surface could have had a smaller impact on this configuration, resulting in a smaller Stark shift.

At higher potentials, the red shift with decreasing coverage was observed next to a broadening of the peak from 550 to 750 mV_RHE_, which needs further investigation. A Fano-type asymmetric peak was observed at the same position as CO_linear_ due to the off-resonance coupling between the resonance of the low-frequency array and the vibrational mode of CO_linear_.^37^ Interestingly, the area of the CO_linear_ peak slightly decreased from 650 to −50 mV_RHE_, while the area of the CO_bridge_ peak showed a continuous increase. Assuming that the area or intensity of the CO peaks is linearly related to the coverage of CO_linear_ and CO_bridge_, the change in peak areas with the voltage suggests an increase in CO_bridge_ coverage and a decrease in CO_linear_ coverage as the cathodic potential increases. Note, however, that dynamic dipole coupling between CO_linear_ and CO_bridge_ leads to an energy transfer from CO_bridge_ to CO_linear_ and thus might lead to deviations from the simple linear relationship between peak areas and coverage.^26^ According to some authors,^29,36^ the competition between CO and hydrogen adsorption on Pt in the cathodic region could have been responsible for a transition from CO_linear_ to CO_bridge_ configurations. Furthermore, the barrier for CO diffusion from a top site to a bridge site was theoretically predicted to be very small.^38^ These findings can explain the increase in the area of the peak attributed to CO_bridge_ to the detriment of the corresponding CO_linear_ peak area, which decreases.

To conclude, our multi-band nanoslot metasurface selectively and simultaneously enhanced and detected with a high accuracy the behavior of CO_linear_ and CO_bridge_. This result appears particularly remarkable, considering the difficulty in detecting the behavior of CO_bridge_ in the literature.^18,19^ In the next section, the study focuses on the behavior of adsorbed CO during the CO_2_ reduction reaction.

### Reduction of CO_2_

**Figure 4:**
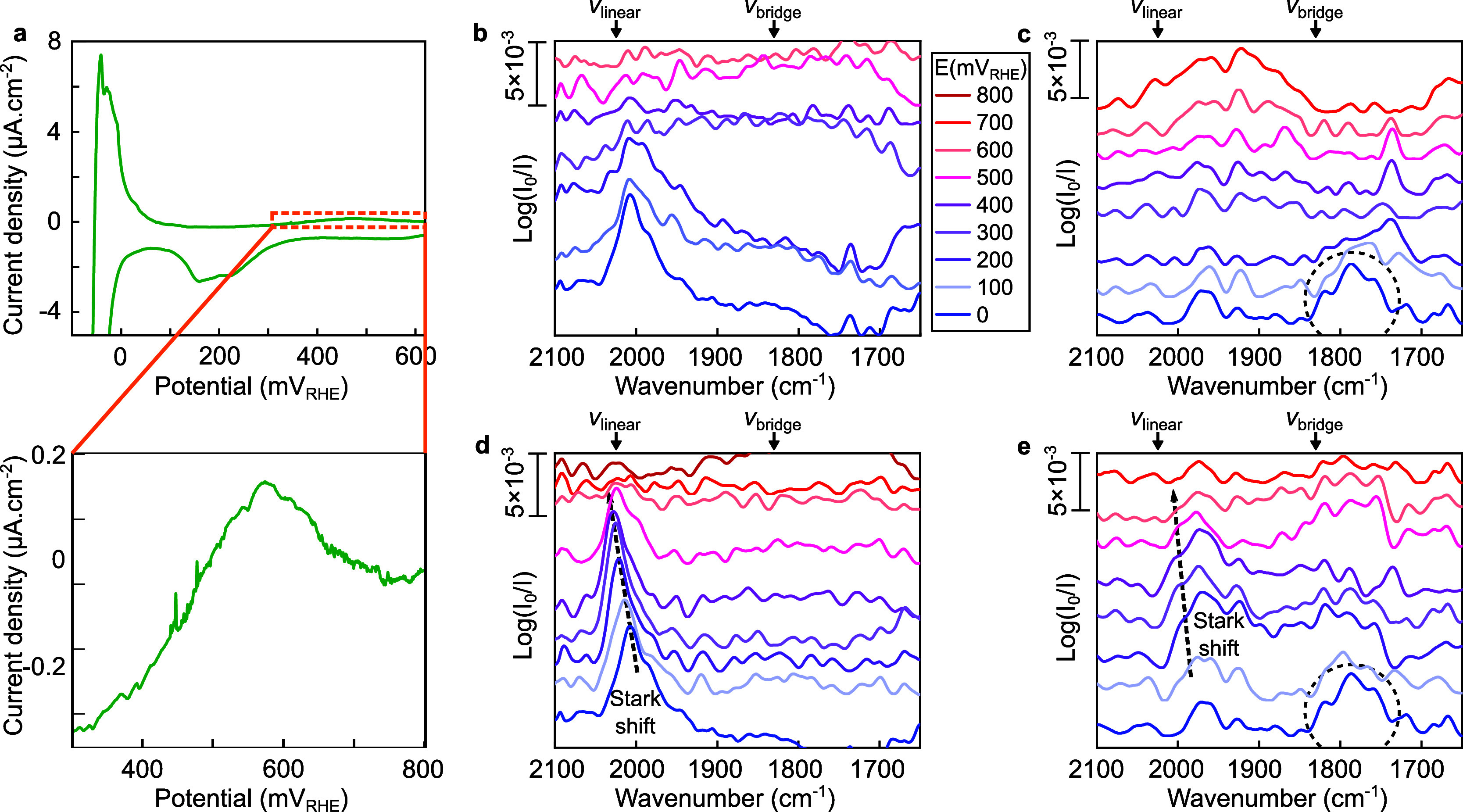
Cyclic voltammetry of the multiband metasurface in 0.5 M K_2_CO_3_ saturated with CO_2_. (a) Evolution of the current density during polarization at 0.25 mV s^–1^. A zoom-out of the current density is shown. (b, c) Evolution of IR spectra with the potential acquired on the (b) high-frequency array optimized for CO_linear_ and (c) low-frequency array optimized for CO_bridge_ detection during the cathodic scan. (d, e) Evolution of IR spectra with the potential acquired on the (d) high-frequency array optimized for CO_linear_ and (e) low-frequency array optimized for CO_bridge_ detection during the anodic scan. The spectral positions of CO_linear_ (ν_linear_) and CO_bridge_ (ν_bridge_) are indicated.

The cathodic scan (Figure 4a) of the cyclic voltammetry of the multiband metasurface in 0.5 M K_2_CO_3_ saturated with CO_2_ shows a dip around 250 mV_RHE_, which is attributed to CO_2_ reduction.^22^ The drop in the current starting at 0 mV_RHE_ is due to the hydrogen evolution reaction and the positive current peak observed after reversing the scan direction stems from the hydrogen oxidation reaction.^28^ Finally, a small oxidative peak was observed around 550 mV_RHE_ and is attributed to the oxidation of the previously formed CO.^39^ Looking at the IR spectra of the high-frequency array optimized for CO_linear_ during the cathodic scan (Figure 4b), a peak was observed around 200 mV_RHE_. This peak indicates the presence of adsorbed CO, which is directly correlated with the reduction peak observed in the voltammogram. Moreover, this peak became more pronounced and more defined with higher cathodic polarizations.

Moving to the low-frequency array optimized for CO_bridge_ detection, at the highest applied cathodic polarization (0 mV_RHE_), a small peak became discernible at around 1785 cm^–1^ in the IR spectra (Figure 4c). This peak is hardly above the detection limit despite the high resolution achieved with our multiband nanophotonic-electrochemical platform and appeared at significantly higher cathodic potentials (200 mV difference) than the CO_linear_ peak obtained with the high-frequency array. Some authors^19,40^ have suggested that the favorable configuration of adsorption of CO on Pt is CO_linear_. Moreover, the literature suggests that CO_bridge_ formation occurs once the CO coverage approaches its maximum limit where a transfer from CO_linear_ to CO_bridge_ takes place.^29,36^

During the anodic scan, the CO_linear_ peak maintained a constant amplitude (Figure 4d) but exhibited a classic Stark shift between 0 to 400 mV_RHE_ followed by an intensity decrease and a small red shift attributed to reduced coverage around 500 mV_RHE_. Then, the peak disappeared, which can be directly correlated to the oxidation peak observed in the voltammogramm (Figure 4a). In contrast, the peak attributed to CO_bridge_ is slightly discernible at 0 mV_RHE_ (Figure 4e). The distortion of the baseline at around 2000 cm^–1^ is attributed to the strong off-resonance coupling between the metasurface-driven resonance of the low-frequency array and the vibrational mode of CO_linear_.^37^ Owing to the high resolution and signal-enhancing properties provided by our nanophotonic-electrochemical platform, Figure 4 suggests that the coverage of CO_bridge_ was low during the reduction of CO_2_ in the alkaline electrolyte. The difference in the results obtained in the CO-saturated electrolyte compared to the CO_2_-saturated electrolyte can likely be attributed to a reduced overall adsorption of CO during the CO_2_RR in the alkaline electrolyte. Specifically, the area under the CO_linear_ peaks during the CO_2_RR was roughly half the size of the area obtained when the electrolyte was saturated with pure CO. The literature^29,36^ indicates that the formation of CO_bridge_ occurs when the coverage of CO approaches its maximum limit. Then, a transfer from CO_linear_ to CO_bridge_ takes place.

Linearly adsorbed and bridge-site adsorbed CO were detected during both CO saturation and oxidation, suggesting that our platform can successfully detect both adsorption configurations if they are present. During the electrochemical reduction of CO_2_, we detected linearly adsorbed CO, evidencing that the platform was working. However, bridge-site adsorbed CO was not observed, suggesting that CO_bridge_ is not significantly involved during the electrochemical reduction of CO_2_ on Pt in K_2_CO_3_. This finding showcases the tremendous potential of our multiband nanophotonic-electrochemical platform to simultaneously characterize multiple adsorbed species in situ during electrochemical reactions.

## Conclusions

We developed a multiband nanophotonic-electrochemical platform enabling enhanced simultaneous in situ characterization of two adsorption configurations of CO on Pt during the electrochemical reduction of CO_2_. Our platform provided a strong enhancement over conventional systems (Pt film with uncontrolled surface morphology) by a factor of over 40. Crucially, our platform was able to detect the CO_bridge_ configuration, which could not be detected here with an unstructured Pt film. Using a straightforward and easily reproducible methodology, we numerically modeled, fabricated, and tested our platform. The CO_linear_ and CO_bridge_ configurations were characterized in CO-saturated electrolyte, suggesting a transition from top to bridge-site configurations at high cathodic potentials and demonstrating the high resolution provided by our platform. The vibrational modes of CO were confirmed via their typical Stark shifts. Our final experimental tests focused on the characterization of the CO_2_RR. Interestingly, we found that during the CO_2_RR in an alkaline environment, despite the high resolution achieved by our platform, the CO_bridge_ configuration was not significantly detected. In contrast, the CO_linear_ configuration was successfully detected. Since bridge-site adsorbed CO was detected during CO saturation and oxidation but not during CO_2_ reduction, we concluded that CO_bridge_ is not significantly involved during the electrochemical reduction of CO_2_ on Pt in K_2_CO_3_. We anticipate that our multi-band nanophotonic-electrochemical platform provides a new strategy to study electrochemical reactions with low coverage or transient features by providing a higher resolution than conventional systems for now limitless IR-active vibrational modes.

## Abbreviations

CO_2_RR: CO_2_ reduction reaction
SEIRAS: surface-enhanced IR absorption spectroscopy
ATR: attenuated total internal reflectance
OCP: open-circuit potential
